# Comparison of risk profiles for new-onset atrial fibrillation between patients aged <60 and ≥60 years

**DOI:** 10.1371/journal.pone.0258770

**Published:** 2021-11-18

**Authors:** In-Soo Kim, Yeon-Jik Choi, Eui-Young Choi, Pil-Ki Min, Young Won Yoon, Byoung Kwon Lee, Bum-Kee Hong, Se-Joong Rim, Hyuck Moon Kwon, Jong-Youn Kim

**Affiliations:** 1 Cardiology, Heart Center, Gangnam Severance Hospital, Yonsei University College of Medicine, Seoul, Republic of Korea; 2 Division of Cardiology, Department of Internal Medicine, Eunpyeong St. Mary’s Hospital, Catholic University College of Medicine, Seoul, Republic of Korea; Policlinico Casilino, ITALY

## Abstract

**Background:**

Atrial fibrillation (AF) has a heterogeneous pathophysiology according to individual patient characteristics. This study aimed to identify the effects of widely known risk factors on AF incidence according to age and to elucidate the clinical implications of these effects.

**Methods and results:**

We analyzed data from 501,668 subjects (≥18years old) without AF and valvular heart disease from the Korean National Health Insurance Service-National Sample Cohort. The total population was divided into two groups according to age, <60years and ≥60years. AF occurred in 0.7% of the overall population (3,416 of 501,668) during the follow-up period (mean 47.6 months). In Cox regression analysis, age, male sex, previous ischemic stroke, heart failure, and hypertension were related to increased risk of new-onset AF in both age groups. Especially in the <60years age group, risk of new-onset AF was increased by relatively modifiable risk factors: obesity (body mass index ≥25kg/m^2^; hazard ratio[HR] 1.37 [1.22–1.55], p<0.001, interaction p<0.001), and hypertension (HR 1.93[1.69–2.22], p<0.001, interaction p<0.001). Although interactions were not significant, chronic obstructive pulmonary disease (HR 1.41[1.24–1.60], p<0.001) and chronic kidney disease (HR 1.28[1.15–1.41], p<0.001) showed increased trends of the risk of new-onset AF in the ≥60years age group.

**Conclusion:**

The risk profile for new-onset AF was somewhat different between the <60years and the ≥60years age groups. Compared to the ≥60years group, relatively modifiable risk factors (such as obesity and hypertension) had a greater impact on AF incidence in the <60years age group. Different management strategies to prevent AF development according to age may be needed.

## Introduction

Atrial fibrillation (AF) is the most common sustained cardiac arrhythmia that occurs in about 1–2% of the general population, and AF is associated with increased morbidity and mortality [1]. The number of patients with AF is predicted to increase substantially in the coming years [1]. The pathophysiologic mechanisms for the development of AF are complex and vary among individuals. A number of studies have attempted to discover risk factors for AF, and the understanding of risk factors has been well established. However, understanding how these factors interact with one another and influence the pathogenesis of AF in an individual remains a challenge [2, 3].

In general, the incidence and the prevalence of AF increase with advancing age [4]. Age is one of the most powerful risk factors for AF [5]. Therefore, comprehension of the interaction between age and other risk factors for AF is critical for assessing an individual’s risk for AF development [6]. In a previous study to identify and characterize the impact of age on national AF hospitalization patterns, younger AF patients were healthier with a different distribution of risk factors than older patients who had higher morbidity and mortality [7]. The objective of our study was to identify the effects of widely known risk factors for AF incidence according to age in the general population and to elucidate the clinical implications of these effects.

## Subjects and methods

### Source of study data

The study population was drawn from the Korean National Health Insurance Service-National Sample Cohort (NHIS-NSC) database. This is a public database, formed by the National Health Insurance Service (NHIS), that includes a 2.2% sample of the population of South Korea [8]. All data and materials have been made publicly available at the National Health Insurance Data Sharing Service and can be accessed at the homepage of the NHIS (https://nhiss.nhis.or.kr/bd/ab/bdaba000eng.do). This sample cohort was extracted by probability sampling, not by a randomized sampling method, from all beneficiaries of the National Health Insurance and National Medical Aid based on the entirety of the national cohort data. Systematic sampling was acquired from each of the 1,476 strata based on age, sex, eligibility status, socioeconomic status and income level. The sample size was proportionate to the cohort size of the strata. The sample’s representativeness was examined previously by comparing the sample to the entire Korean population [9, 10]. The subjects’ disease information was classified according to the 10^th^ revision of the International Classification of Disease (ICD-10) codes from the primary care institution and the secondary and tertiary hospitals.

The Sample Cohort database consisted of three datasets: (i) sociodemographic information of the beneficiaries; (ii) medical claims including information on diagnoses based on the ICD-10 codes, outpatient (including individual physician office) visits, admissions, and treatments; and (iii) National Health Examination data of the cohort members. Begun in 2002, the National Health Examination dataset was created for the entire Korean population by the National Health Insurance Corporation. The death registration database of the Korea National Statistical Office, which includes the date and cause of death, was linked with the NHIS cohort database [9, 10].

This study was approved by the Institutional Review Board (IRB) of Yonsei University College of Medicine in Seoul, Korea. This study was based on NHIS data that was completely anonymized and de-identified for analysis. The IRB waived the requirement for informed consent, and this study was conducted under the tenets of the Declaration of Helsinki.

### Study population

From the Korean National Health Insurance Service-National Sample Cohort (NHIS-NSC), a total of 506,805 subjects aged >18 years old who received a medical check-up between January 1, 2009 and December 31, 2015 were enrolled in this study. Patients who had mitral stenosis or prosthetic valve disease (n = 596) or patients with prevalent AF (n = 4,541) were excluded. Mitral stenosis or prosthetic valve disease was defined from any diagnoses of mitral stenosis or heart valve surgery (ICD-10 codes: I05.0, I05.2, I34.2, Z95.2–4), and prevalent AF was defined from diagnosis with previous insurance claim for AF (I48) within 7 years (2002–2008: disease-free baseline period) (S1 Table in S1 File). After exclusion, a total of 501,668 patients, including 392,332 patients who were younger than 60 years old (“aged <60 years” group) and 109,336 patients who were older than 60 years old (“aged ≥60 years” group) were enrolled in this study (S1 Fig in S1 File).

### Risk factors of AF

We considered these variables as risk factors for AF: age, male sex, body mass index (BMI), previous ischemic stroke or transient ischemic attack (TIA), previous myocardial infarction (MI), heart failure, hypertension, diabetes, chronic pulmonary obstructive disease (COPD), chronic kidney disease (CKD), obesity, smoking, excessive alcohol intake, and low physical activity [1]. Obesity, smoking, excessive alcohol intake, and low physical activity are considered modifiable risk factors; the others are considered non-modifiable risk factors.

The definitions of comorbidities are presented in S1 Table in S1 File. Obesity is defined as a BMI equal to or greater than 25 kg/m^2^ using the World Health Organization (WHO) Asia-Pacific classification. Current or former smokers with at least a 1 pack-year history of smoking were assigned the smoking risk factor. Excessive alcohol intake was defined as the consumption of more than 14 glasses per week for men and more than 7 glasses per week for women [11]. Low physical activity was defined as a weekly level of moderate- to vigorous-intensity physical activities less than 500 MET-minutes/week [12].

### Outcomes

AF was diagnosed using ICD-10 codes (ICD-10: I48). To ensure accurate diagnosis, we defined patients with AF only when AF was a discharge diagnosis or was confirmed more than twice in the outpatient department. Diagnosis of AF has previously been validated in the NHIS database with a positive predictive value of 94.1% [13, 14]. All outcomes were analyzed in the “aged <60 years” and “aged ≥60 years” groups.

### Statistical analysis

Data are presented as mean ± standard deviation (SD) for continuous variables and as proportions for categorical variables. Continuous variables were compared using the Student’s t-test. Analysis of categorical variables was performed with Fisher’s exact test [15]. Cox-proportional hazard regression analysis was performed to estimate the adjusted hazard ratio (HR) for the association between risk factors and new-onset AF. Since mortality is the major competing risk for the cardiovascular outcomes (in this study, incident AF), we also analyzed the Fine and Gray regression model considering death as a competing risk (S1 File) to estimate the adjusted subdistribution hazard ratio (sHR) for new-onset AF [16, 17]. To analyze interaction tests for each risk factor between “aged <60 years” and “aged ≥60 years” groups, we used “(whether age≥60 years or not) * (each confounder)” terms while adjusting for other residual covariables. The model used these variables that were a priori considered to be important risk factors for AF: age, sex, previous ischemic stroke or TIA, previous MI, heart failure, hypertension, diabetes mellitus, COPD, CKD, obesity, smoking, excessive alcohol intake, and low physical activity. All independent variables were entered into the model. The relationship between BMI, smoking frequency, and the incidence of AF is graphically illustrated with smooth curves fitted to the Cox proportional hazards model using a penalized B-spline method. The splines between BMI, smoking frequency, and HR of new-onset AF were adjusted for the relevant covariates. To investigate the association between alcohol intake and incident AF, we also performed Cox regression analysis with alcohol intake as the continuous variable.

We analyzed the linear estimates of HRs concerning the relationship between age and incident AF and tested these estimates using the log-linear model with a thin plate spline. The result of this analysis was that incident AF increased at age > 59.8 years (S2 Fig in S1 File). Based on this result, to acquire the specific age cut-off at which incident AF was increased, we also analyzed the sensitivities and specificities of different age ranges and determined the point with the highest Youden’s index (sensitivity + specificity—1) [18]. This analysis consistently showed a cut-off range of age ≥60 years (S2 Table in S1 File) as having the highest Youden’s index. Also, we estimated the predictive accuracy of this age cut-off (analyzed by the highest Youden’s index from different age ranges) for incident AF by calculating the c-index on the basis of the receiver operating characteristic curve from logistic regression models [19]. The c-index for age ≥60 years in predicting incident AF was 0.81 (S2 Fig in S1 File). Then, we divided the population into two groups, “aged <60 years” and “aged ≥60 years”.

All tests were two-tailed with a p-value <0.05 considered as significant. Statistical analyses were conducted with SPSS version 23.0 statistical package (SPSS Inc., Chicago, IL, USA), and R statistical software, version 3.6.0 (R Foundation for Statistical Computing, Vienna, Austria).

## Results

### Baseline characteristics

In the overall population, the mean age was 47.6 ± 14.3 years old, 50% of patients were male, and the mean CHA_2_DS_2_-VASc score was 1.1 ± 1.2 (Table 1). During follow-up periods of 46.2 ± 15.1 months, there were 3,416 of 501,668 (0.68%) new-onset AF cases. 1,189 cases (0.24%) of new-onset AF were in the “aged <60 years” group and 2,227 cases (0.44%) were in the “aged ≥60 years” group. As anticipated, the rate of chronic and severe comorbidities including heart failure, hypertension, diabetes mellitus, CKD, COPD, previous ischemic stroke or TIA, and previous MI was higher in the “aged ≥60 years” group than the “aged <60 years” group (all p-values <0.001).

**Table 1:** Baseline characteristics.

	Overall (n = 501,668)	“Aged <60 years” group (n = 392,332)			“Aged ≥60 years” group (n = 109,336)		
		AF-free (n = 391,143)	Incident AF (n = 1,189)	p value	AF-free (n = 107,109)	Incident AF (n = 2,227)	p value
Age, years old	47.6 ± 14.3	41.9 ± 10.1	48.7 ± 8.6	<0.001	68.0 ± 6.4	70.8 ± 6.8	<0.001
Male	251,055 (50.0%)	200,808 (51.3%)	778 (65.4%)	<0.001	48,250 (45.0%)	1,219 (54.7%)	<0.001
Follow-up duration, months	46.2 ± 15.1	46.1 ± 15.4	50.9 ± 11.6	<0.001	46.7 ± 14.1	48.7 ± 13.0	<0.001
CHA_2_DS_2_-VASc score	1.1 ± 1.2	0.7 ± 0.8	1.0 ± 1.1	<0.001	2.4 ± 1.6	3.1 ± 1.7	<0.001
Heart failure	10,851 (2.2%)	3170 (0.8%)	56 (4.7%)	<0.001	7266 (6.8%)	359 (16.1%)	<0.001
Hypertension	107,833 (21.5%)	47,371 (12.1%)	422 (35.5%)	<0.001	58,471 (54.6%)	1,569 (70.5%)	<0.001
Diabetes mellitus	31,388 (6.3%)	13,443 (3.4%)	123 (10.3%)	<0.001	17,367 (16.2%)	455 (20.4%)	<0.001
CKD	29,259 (5.8%)	11,848 (3.0%)	67 (5.6%)	<0.001	16,788 (15.7%)	556 (25.0%)	<0.001
COPD	11,442 (2.3%)	3,728 (1.0%)	21 (1.8%)	0.006	7,411 (6.9%)	282 (12.7%)	<0.001
Dyslipidemia	96,608 (19.3%)	52,820 (13.5%)	354 (29.8%)	<0.001	42,438 (39.6%)	996 (44.7%)	<0.001
Chronic liver disease	112,036 (22.3%)	77,301 (19.8%)	393 (33.1%)	<0.001	33,531 (31.3%)	811 (36.4%)	<0.001
Previous ischemic stroke / TIA	18,466 (3.7%)	5,446 (1.4%)	58 (4.9%)	<0.001	12,547 (11.7%)	415 (18.6%)	<0.001
Previous MI	4,532 (0.9%)	1,684 (0.4%)	23 (1.9%)	<0.001	2,723 (2.5%)	102 (4.6%)	<0.001
History of cancer	33,378 (6.7%)	17,638 (4.5%)	108 (9.1%)	<0.001	15,249 (14.2%)	383 (17.2%)	<0.001
Obesity (BMI > 25 kg/m^2^)	157,989 (31.5%)	118,385 (30.3%)	536 (45.1%)	<0.001	38,248 (35.7%)	820 (36.8%)	0.284
BMI, kg/m^2^	23.7 ± 3.3	23.6 ± 3.3	24.7 ± 3.4	<0.001	24.0 ± 3.2	24.1 ± 3.4	0.178
Smoking, current or former	186,532 (37.2%)	154,488 (39.5%)	584 (49.1%)	<0.001	30,719 (28.7%)	741 (33.3%)	<0.001
Smoking amount, pack-year	6.0 ± 11.8	5.6 ± 10.3	9.5 ± 14.4	<0.001	7.6 ± 15.8	9.2 ± 17.5	<0.001
Excessive alcohol intake	89,743 (17.9%)	78,710 (20.1%)	265 (22.3%)	0.065	10,529 (9.8%)	239 (10.7%)	0.161
Alcohol amount, glass / week	7.0 ± 14.1	7.7 ± 14.4	9.6 ± 17.4	<0.001	4.3 ± 12.6	5.0 ± 14.7	0.032
Low physical activity	385,346 (76.8%)	301,409 (77.1%)	894 (75.2%)	0.129	81,371(76.0%)	1,702 (76.4%)	0.634
MET-minutes/week	284.1 ± 416.0	281.8 ± 397.4	311.8 ± 425.9	0.016	292.5 ± 476.5	278.7 ± 466.5	0.177
Systolic BP, mmHg	122.2 ± 15.3	120.3 ± 14.5	124.7 ± 15.7	<0.001	129.2 ± 16.0	130.5 ± 17.2	<0.001
Diastolic BP, mmHg	76.1 ± 10.2	75.5 ± 10.2	78.3 ± 10.5	<0.001	78.1 ± 10.1	78.9 ± 10.7	0.002
Hemoglobin, g/dL	13.9 ± 1.6	14.0 ± 1.7	14.3 ± 1.7	<0.001	13.5 ± 1.5	13.6 ± 1.6	0.006
Fasting blood glucose, mg/dL	97.8 ± 24.8	96.1 ± 23.3	103.4 ± 33.6	<0.001	104.2 ± 28.6	106.0 ± 31.4	0.005
Total cholesterol, mg/dL	194.9 ± 37.3	194.0 ± 36.6	193.5 ± 38.3	0.677	198.6 ± 39.3	191.8 ± 40.3	<0.001
Serum creatinine, mg/dL	1.0 ± 1.1	1.0 ± 1.2	1.1 ± 1.3	0.009	1.0 ± 1.0	1.1 ± 1.1	<0.001

Values are expressed as n (%) or mean ± standard deviation. AF, atrial fibrillation; BMI, body mass index; BP, blood pressure; CKD, chronic kidney disease; COPD, chronic obstructive pulmonary disease; MET, metabolic equivalent of task; MI, myocardial infarction; TIA, transient ischemic attack.

The comparisons of baseline characteristics between AF-free and incident AF patients are presented in Table 1. Age, male sex, comorbidities, and smoking were significantly higher in incident AF patients than AF-free patients in both the “aged <60 years” and “aged ≥60 years” groups (Table 1). In the “aged <60 years” group, obesity of incident AF patients was higher than that of AF-free patients, but there was no difference in the “aged ≥60 years” group. There was no difference in excessive alcohol intake and low physical activity between AF-free and incident AF patients in both the “aged <60 years” and “aged ≥60 years” groups.

### The crude incidence rate of new-onset AF according to risk factors

Table 2 shows the crude incidence rate of new-onset AF according to risk factors in the “aged <60 years” and “aged ≥60 years” groups. Regarding all non-modifiable risk factors such as male sex, previous ischemic stroke or TIA, previous MI, diagnosed heart failure, hypertension, diabetes mellitus, COPD, and CKD, patients with a certain risk factor had a higher incidence of new-onset AF than patients without the risk factor in both the “aged <60 years” and “aged ≥60 years” groups. In the “aged <60 years” group, obese patients had a higher incidence of new-onset AF than non-obese patients (1.17 vs. 0.62 per 1,000 person-years, p<0.001). However, in the “aged ≥60 years” group, there was no significant difference in new-onset AF incidence between obese and non-obese patients (5.41 vs. 5.21 per 1,000 person-years, p = 0.391). Patients who smoked had a higher incidence of new-onset AF than never-smokers in both the “aged <60 years” (0.98 vs. 0.67 per 1,000 person-years, p<0.001) and “aged ≥60 years” groups (6.20 vs. 4.92 per 1,000 person-years, p<0.001). Regardless of age group, there was no significant difference in the incidence of new-onset AF according to excessive alcohol intake and low physical activity. Some variables (eGFR [ml/min], BMI [kg/m^2^], smoking [pack∙year], alcohol intake [g/week], and physical activity habits [hours/week]) were analyzed as continuous variables as shown in S3 Table in S1 File.

**Table 2:** The incidence rates of new-onset AF according to risk factors in the “aged <60 years” and “aged ≥60 years” groups.

New-onset AF	“Aged <60 years” group (n = 392,332)				“Aged ≥60 years” group (n = 109,336)			
	Number of events	Person-years	Incidence rate* (95% CI)	p-value	Number of events	Person-years	Incidence rate* (95% CI)	p-value
**Non-modifiable risk factors**								
**Sex**								
Female	411	716,313	0.57 (0.52–0.63)	<0.001	1,008	231,155	4.36 (4.09–4.63)	<0.001
Male	778	788,824	0.99 (0.92–1.06)		1,219	190,747	6.39 (6.03–6.75)	
**Previous ischemic stroke or TIA**								
No	1,131	1,484,606	0.76 (0.72–0.81)	<0.001	1,812	374,248	4.84 (4.62–5.06)	<0.001
Yes	58	20,530	2.83 (2.10–3.55)		415	47,654	8.71 (7.87–9.55)	
**Previous MI**								
No	1,166	1,498,822	0.78 (0.73–0.82)	<0.001	2,125	411,740	5.16 (4.94–5.39)	<0.001
Yes	23	6,314	3.64 (2.31–5.47)		102	10,163	10.0 (8.18–12.2)	
**Diagnosed heart failure**								
No	1,133	1,493,141	0.76 (0.71–0.80)	<0.001	1,868	394,334	4.74 (4.52–4.95)	<0.001
Yes	56	11,996	4.67 (3.45–5.89)		359	27,568	13.0 (11.7–14.4)	
**Diagnosed hypertension**								
No	767	1,321,844	0.58 (0.54–0.62)	<0.001	658	193,842	3.39 (3.14–3.65)	<0.001
Yes	422	183,292	2.30 (2.08–2.52)		1,569	228,060	6.88 (6.54–7.22)	
**Diagnosed diabetes**								
No	1,066	1,454,461	0.73 (0.69–0.78)	<0.001	1,772	355,790	4.98 (4.75–5.21)	<0.001
Yes	123	50,675	2.43 (2.00–2.86)		455	66,112	6.88 (6.25–7.51)	
**Diagnosed COPD**								
No	1,168	1,491,435	0.78 (0.74–0.83)	0.002	1,945	393,931	4.94 (4.72–5.16)	<0.001
Yes	21	13,702	1.53 (0.88–2.19)		282	27,972	10.1 (8.91–11.3)	
**Diagnosed CKD**								
No	1,122	1,453,907	0.77 (0.73–0.82)	<0.001	1,671	356,372	4.69 (4.46–4.91)	<0.001
Yes	67	50,950	1.32 (1.00–1.63)		556	65,355	8.51 (7.80–9.21)	
**Modifiable risk factors**								
**Obesity (BMI > 25 kg/m** ^**2**^ **)**								
No	653	1,048,816	0.62 (0.57–0.67)	<0.001	1,407	270,238	5.21 (4.93–5.48)	0.391
Yes	536	456,321	1.17 (1.08–1.27)		820	151,664	5.41 (5.04–5.78)	
**Smoking, current or former**								
No	605	908,032	0.67 (0.61–0.72)	<0.001	1,486	302,305	4.92 (4.67–5.17)	<0.001
Yes	584	597,104	0.98 (0.90–1.06)		741	119,598	6.20 (5.75–6.64)	
**Excessive alcohol intake**								
No	924	1,203,218	0.77 (0.72–0.82)	0.055	1,988	380,300	5.23 (5.00–5.46)	0.184
Yes	265	301,919	0.88 (0.77–0.98)		239	41,602	5.74 (5.02–6.47)	
**Low physical activity**								
No	295	351,746	0.84 (0.82–1.02)	0.240	525	103,294	5.08 (4.65–5.54)	0.319
Yes	894	1,153,391	0.78 (0.73–0.83)		1,702	318,608	5.34 (5.09–5.60)	

*Crude incidence rates are presented as incidence per 1,000 person-years (95% CI). AF, atrial fibrillation; BMI, body mass index; BP, blood pressure; CI, confidence interval; CKD, chronic kidney disease; COPD, chronic obstructive pulmonary disease; MET, metabolic equivalent of task; MI, myocardial infarction; TIA, transient ischemic attack.

### Risk factors of new-onset AF according to age group

In the overall population, age, male sex, age, male sex, previous ischemic stroke or TIA, heart failure, hypertension, diabetes mellitus, COPD, CKD, obesity, and smoking were associated with increased risk of new-onset AF (S3 Fig in S1 File). To investigate the robustness of our analysis, we additionally performed a multivariable adjusted regression analysis using age as a categorical variable (“age ≥60 years or not”), and the result was consistent with our previous analysis (S4 Table in S1 File).

**Fig 1:**
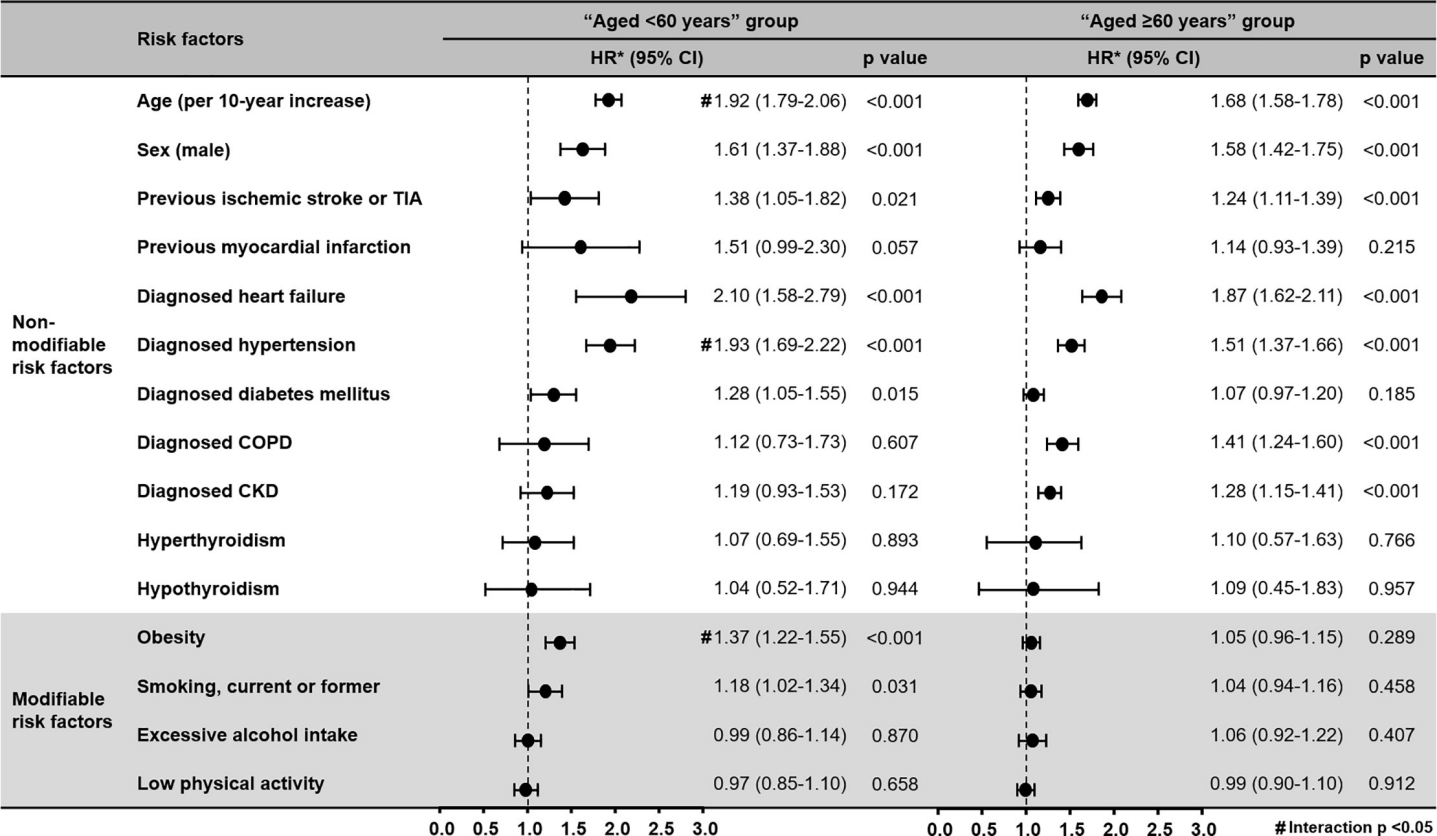
Hazard ratios for new-onset AF according to the risk factors in the “aged <60 years” and “aged ≥60 years” groups. * Hazard ratio for each risk factor was adjusted by the Cox regression model using these variables: age, sex, previous ischemic stroke or TIA, previous myocardial infarction, heart failure, hypertension, diabetes mellitus, COPD, CKD, obesity, smoking, excessive alcohol intake, and low physical activity. # Indicates interaction p-values <0.05 in testing for interactions with each risk factor between “aged <60 years” and “aged ≥60 years” groups while adjusting for other residual covariables (age, sex, previous ischemic stroke/TIA, previous myocardial infarction, heart failure, hypertension, diabetes mellitus, COPD, CKD, obesity, smoking, excessive alcohol intake, and low physical activity). Among subjects aged <60 years, the risks of incident AF were significantly higher for age (per 10-year increases), hypertension, and obesity (each interaction p-value <0.001) compared to those of subjects aged ≥60 years. CI, confidence interval; CKD, chronic kidney disease; COPD, chronic obstructive pulmonary disease; HR, hazard ratio; TIA, transient ischemic attack.

Analysis of risk factors for new-onset AF according to age group is presented in Fig 1. Age, male sex, previous ischemic stroke or TIA, heart failure, and diabetes were associated with increased risk of new-onset AF in both age groups. In the “aged <60 years” group, risk of new-onset AF was increased by hypertension (HR 1.93, 95% confidence interval [CI] 1.69–2.22, p<0.001), obesity (HR 1.37, 95% CI 1.22–1.55, p<0.001), and current or former smoking (HR 1.18, 95% CI 1.02–1.34, p = 0.031). In the “aged ≥60 years” group, risk of new-onset AF was increased by COPD (HR 1.41, 95% CI 1.24–1.60, p<0.001) and CKD (HR 1.28, 95% CI 1.15–1.41, p<0.001). Among subjects aged <60 years, the risks of incident AF were significantly higher for age (per 10-year increase), hypertension, and obesity (each interaction p-value <0.001) compared to those of subjects aged ≥60 years (Fig 1). Fine and Gray regression analyses were also performed, and the overall consistent results are shown in S4 and S5 Figs in S1 File.

To investigate the dose-responsive relationship between alcohol intake and the risk of incident AF, another Cox proportional hazard regression analysis was performed with alcohol intake as the continuous variable (not a categorical variable as previously analyzed). Alcohol intake was significantly correlated with the risk of incident AF among both “aged <60 years” (HR 1.05 [1.02–1.07], p<0.001) and “aged ≥60 years” (HR 1.04 [1.02–1.06], p<0.001) groups (interaction p-value between these groups was 0.355). The relationship between alcohol intake and incident AF was tested using the log-linear model with a thin-plate spline, and the results were consistent (S6 Fig in S1 File).

### Effect of BMI and smoking frequency on the risk of new-onset AF according to age group

**Fig 2:**
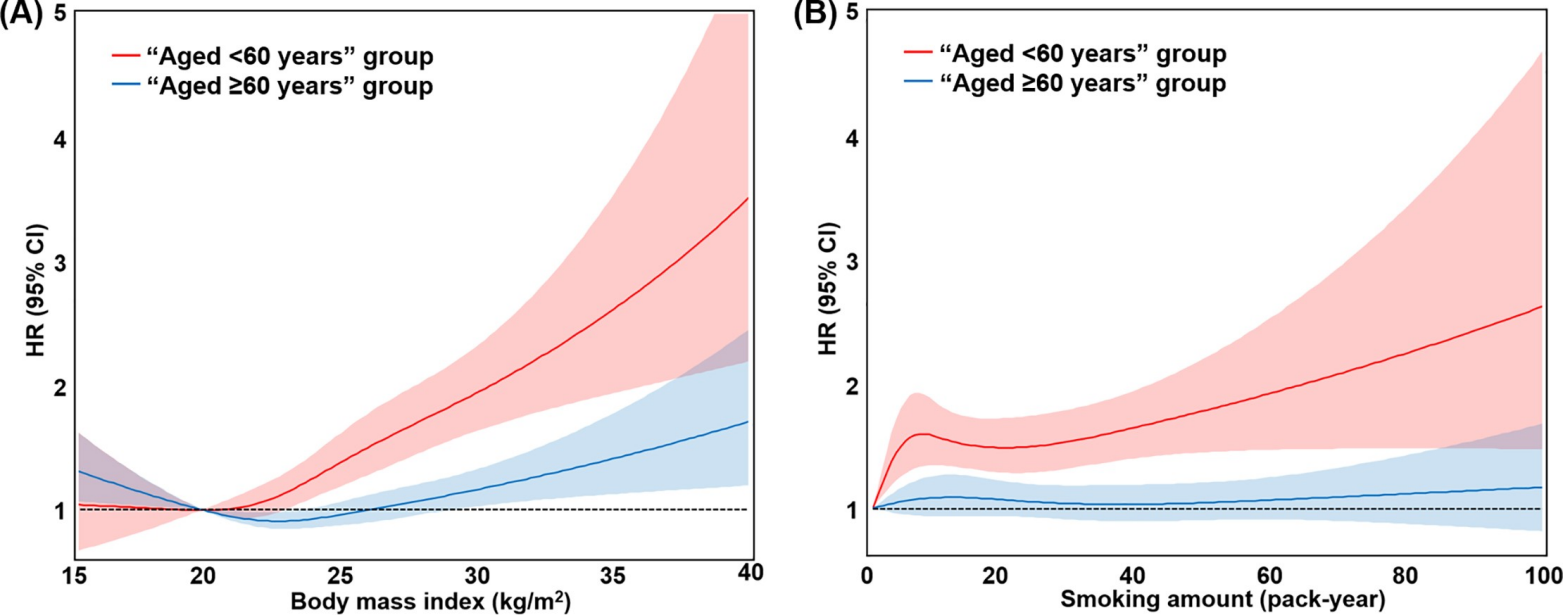
(A) Body mass index and adjusted hazard ratios for new-onset AF in the “aged <60 years” and “aged ≥60 years” groups. (B) Smoking frequency (pack-year) and adjusted hazard ratios for new-onset AF in the “aged <60 years” and “aged ≥60 years” group. Hazard ratio (HR) for each risk factor was adjusted for by the Cox regression analysis using these variables: age, sex, previous ischemic stroke or transient ischemic attack, previous myocardial infarction, heart failure, hypertension, diabetes mellitus, chronic obstructive pulmonary disease, chronic kidney disease, obesity, smoking, excessive alcohol intake, and low physical activity (obesity was excluded in the model for [A], smoking status was excluded in the model for [B]). A reference for hazard ratio was a BMI of 20 kg/m^2^ in (A) and never-smokers in (B). Color areas are 95% confidence intervals for the spline curves. HR, hazard ratio.

The risks of new-onset AF associated with BMI and smoking frequency are presented using restricted cubic spline analysis in Fig 2. In the “aged <60 years” group, the risk of new-onset AF increased with increase in BMI. However, a non-linear U-shaped association was found between BMI and AF risk in the “aged ≥60 years” group (Fig 2A). In the “aged <60 years” group, there was a positive correlation between smoking frequency and AF risk. However, there was no association between smoking frequency and AF risk in the “aged ≥60 years” group (Fig 2B). The Fine and Gray competing risk model analyses were also shown to be consistent (S7 Fig in S1 File).

## Discussion

The main findings of this study were that there were differences in the risk factors associated with new-onset AF between the “aged <60 years” and “aged ≥60 years” groups although there were some common risk factors. Compared to the “aged ≥60 years” group, obesity was more associated with an increased risk of new-onset AF in the “aged <60 years” group. Also, smoking showed an increased risk trend in new-onset AF in the “aged <60 years” group. However, COPD and CKD showed increased risk trends in new-onset AF in the “aged ≥60 years” group. Compared to the “aged ≥60 years” group, modifiable risk factors (such as obesity) had greater impact on the risk of new-onset AF in the “aged <60 years” group.

### Differences in the risk factors of new-onset AF according to age

In contrast to the “aged <60 years” group, CKD and COPD had greater associations with increased risk of new-onset AF in the “aged ≥60 years” group. CKD and AF have common risk factors and pathogenic pathways; being elderly a is strong and common risk factor in both diseases [20]. As renal function declines or albuminuria increases with age, proinflammatory biomarkers such as interleukin-6, fibrinogen, and C-reactive protein increase. These markers are risk factors for AF [21]. Also, chronic hypoxia, respiratory acidosis, and cor pulmonale have been suggested to be triggers of atrial arrhythmias and to be major causes for the association between COPD and the increased risk of AF. These chronic illnesses are often considered as irreversible conditions and are usually associated with elderly patients [22]. In the “aged <60 years” group, there was a possibility of modification in the risk factors. Obesity is a modifiable risk factor and its impact is greater in the younger age group [23–25]. A recent Framingham study concerned childhood secondhand smoke exposure. This exposure was thought to be associated with an increased risk for development of AF in younger adults. Smoke exposure had a significant role in the development of AF in a younger age population [26]. The hypothesis was that smoking increases oxidative stress, alters plasma catecholamine concentrations, and accelerates atrial remodeling thereby contributing to AF incidence [27]. Also, thyroid hormones have effects on cardiovascular hemodynamics, metabolism, and tachy-/brady-arrhythmias; both hyperthyroidism and hypothyroidism can affect incident AF [28]. Although our results were not statistically significant, these results showed increased trends between hyperthyroidism and hypothyroidism and incident AF in both age groups (Fig 1). From this perspective, as age increases, cumulative exposures of these factors increase, but their relative effects become less prominent. Unlike CKD and COPD, hypertension is a relatively amendable risk factor. Therefore, if this risk factor is improved, a better prognosis is expected in the “aged <60 years” group compared to the “aged ≥60 years” group.

### Different pathogenesis of AF according to age

Although the incidence and the prevalence of AF increase with age, the fundamental cause of AF development with aging is unclear. This is difficult to study in humans because of the long follow-up period and the substantial number of confounding factors. Nevertheless, we can assume that the pathogenesis of AF is different according to age group because there were differences in the risk factors for new-onset AF in the “aged <60 years” and “aged ≥60 years” groups.[29] Although we did not investigate the family history of the included population, De With et al. [29] suggested that a quarter of young AF patients have a family history of AF and also suggested that most young AF patients had risk factors or comorbidities such as obesity, premature hypertension [30], alcohol consumption [31], vigorous physical activity [32], and hypertrophic cardiomyopathy [33] and congenital heart disease [34]. This is the basis for the recommendation that improving lifestyle choices and behavior modification should be the focus when managing younger AF patients. The exact pathophysiological mechanisms have not been elucidated, and clarifying the pathogenesis of AF across age groups requires additional study.

### The goal of AF management according to age

Although there were some differences in the risk factors for new-onset AF according to age groups, managing known cardiovascular diseases is most important to prevent AF [1]. Our results showed some differences in the risk factors for incident AF across age groups; therefore, different management strategies may be needed for these age groups. In the “aged <60 years” group, because modifiable risk factors, particularly obesity, are thought to have greater impact on incident AF, managing these factors is important in preventing AF development and related adverse outcomes [30]. However, in the “aged ≥60 years” group, because the impact of modifiable risk factors on incident AF is smaller than the impact of chronic risk factors that are difficult to ameliorate, early detection of AF and application of relevant management strategies is necessary. Although managing underlying cardiovascular comorbidities is important to prevent AF development in the general population [1], lifestyle modification, as mentioned earlier, requires emphasis in relatively younger subjects in an effort to manage modifiable risk factors. Since the impact of chronic comorbidities on incident AF are smaller in younger subjects compared to the older subjects, cardiac structural remodeling is thought to be less severe in younger AF patients [25]. The rhythm control strategy with catheter ablation in relatively younger patients with less structural remodeling has better outcomes than in elderly patients. This strategy may be a viable option when managing younger patients [30]. A recent study supported the management of borderline risk factors in relatively younger patients without traditional risk factors for AF [35]. Marcos et al. investigated about sex-based differences in clinical profiles, especially in younger AF patients, but did not find significant differences in cardiovascular outcomes [36]. Siland et al. demonstrated sex-based differences in genetically determined BMI and the risk of AF progression; this group suggested that genetic factors should be included to optimize AF management [37]. This emphasizes that different management strategies according to age are needed to prevent incident AF. However, prospective studies are needed to test this assertion.

### Study limitations

Our study had several limitations. We included subjects from the NHIS-NSC database who had undergone National Health Examinations. This may have resulted in selection bias. However, our database was compiled using a stratified sampling method based on age, sex, eligibility status, socioeconomic status, and income level. Also, the sample’s representativeness was examined previously [9]. Because the information on smoking frequency, alcohol intake, and physical activity were obtained from questionnaires during the National Health Examination, there is the possibility that this information is another source of bias. Second, studies using the NHIS-based administrative claim data are potentially susceptible to errors from disease coding inaccuracies. To minimize this problem, we applied the definitions of the diagnoses that we had already validated in previous studies using the Korean NHIS-NSC. For example, the positive predictive value for the diagnosis of AF using the ICD-10 code (I48) was 94.1% [13, 14, 38, 39]. Third, since we defined AF patients only with the ICD-10 codes, paroxysmal or asymptomatic AF patients may have been missed. Fourth, we did not apply all known AF risk factors in this study. Because our analysis used national health administrative claim data, some risk factors of AF, such as sleep apnea or genetic predisposition, were not included in the analyses. Additionally, careful interpretation is needed because subjects with missing data were more frequent in the “aged ≥60 years” group compared to the “aged <60 years” group (1.9% vs. 0.7%, p = 0.011). However, the number of subjects with missing data was small (less than 2.0%).

Alcohol consumption has been associated with incident AF in previous studies. The mechanism has been suggested to be direct myocardial injury and inflammation and oxidative stress [40]. However, in this study, excessive alcohol intake and low physical activity were not significantly correlated with incident AF (Fig 1). Because physical activity and alcohol intake behavior were obtained from questionnaires during the national health examinations, careful interpretation of our findings is needed. In a previous publication, our group demonstrated a U-shaped dose-response relationship between physical activity and incident AF [12].

## Conclusion

Different trends were shown in the risk factors for incident AF between the “aged <60 years” and “aged ≥60 years” groups. Relatively modifiable risk factors, such as obesity and hypertension, had a greater impact on incident AF in the “aged <60 years” group. This suggests that incident AF can be reduced by weight reduction and hypertension treatment, especially in the “aged <60 years” group. The pathogenesis of AF may have differences according to age. Therefore, different management strategies according to age may be needed to prevent AF development. Prospective studies are needed to test this assertion.

## Supporting information

S1 File (DOCX)
