# Supplementary material for: Comparison of risk profiles for new-onset atrial fibrillation between patients aged <60 and ≥60 years
Source: PLoS One. 2021 Nov 18;16(11):e0258770. doi: 10.1371/journal.pone.0258770 (PMC8601466; doi:10.1371/journal.pone.0258770)
Supplement: S1 File — (DOCX) [file pone.0258770.s001.docx]

**SUPPLEMENTAL ONLINE MATERIAL****S**

**Supporting Information**

Supporting Files – contains all the Supplemental Tables and Figures.

**Supplemental References**

1. Levey AS, Stevens LA, Schmid CH, Zhang YL, Castro AF, 3rd, Feldman HI, et al. A new equation to estimate glomerular filtration rate. Ann Intern Med. 2009;150: 604-612. doi:10.7326/0003-4819-150-9-200905050-00006 PMID:19414839

**Supplemental Figure S1.** Flowchart of the study population. AF, atrial fibrillation; ICD-10, 10^th^ revision of the International Classification of Diseases; NHIS, the National Health Insurance Service


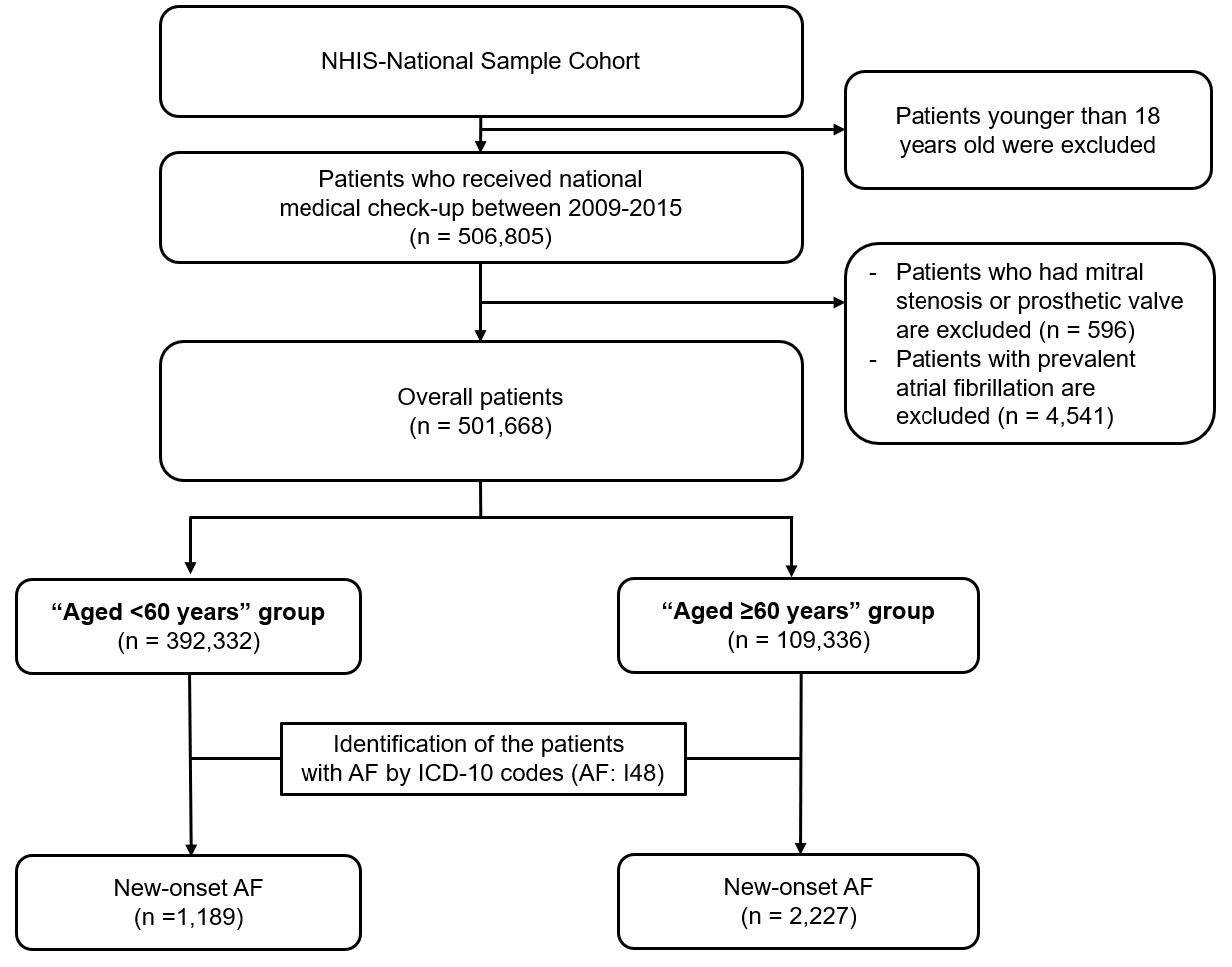


**Supplemental Figure S2.** Age-responsive linear relationship (larger image) between age and the incident AF tested by log-linear model with a thin-plate spline (age, sex, previous ischemic stroke/transient ischemic attack, myocardial infarction, heart failure, hypertension, diabetes, chronic obstructive pulmonary disease, chronic kidney disease, obesity, smoking, excessive alcohol intake, and low physical activity-adjusted HRs). It also showed the specific age cutoff (age >59.8 years: lower 95% CI more than 1.0 of HR) associated with the increased risk of the incident AF. Also, we estimated the predictive accuracy of the age cutoff (analyzed by the highest Youden’s index from different age ranges in Supplemental Table S2) in case of incident AF by calculating the c-index on the basis of the receiver operating characteristic curve from logistic regression models (smaller image). The c-index for age ≥60 years in predicting the incident AF was 0.81. AF, atrial fibrillation; CI, confidence interval; HR, hazard ratio.


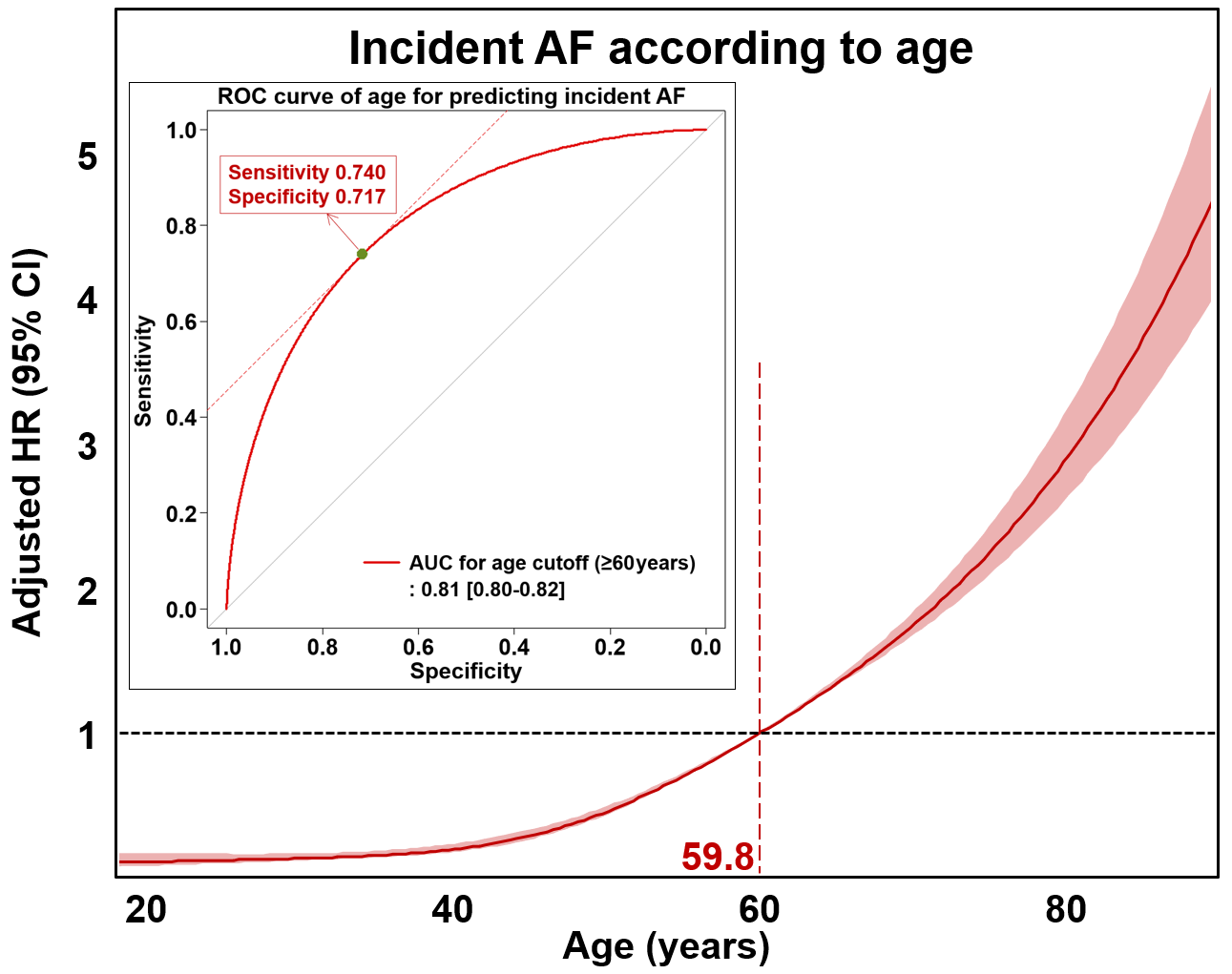


**Supplemental Figure S3.** Hazard ratios for new-onset AF according to the risk factors in the overall population. *Hazard ratio for each risk factor was adjusted by the Cox regression model using these variables: age, sex, previous ischemic stroke or TIA, previous myocardial infarction, heart failure, hypertension, diabetes mellitus, COPD, CKD, obesity, smoking, excessive alcohol intake, and low physical activity. CI, confidence interval; CKD, chronic kidney disease; COPD, chronic obstructive pulmonary disease; HR, hazard ratio; TIA, transient ischemic attack.


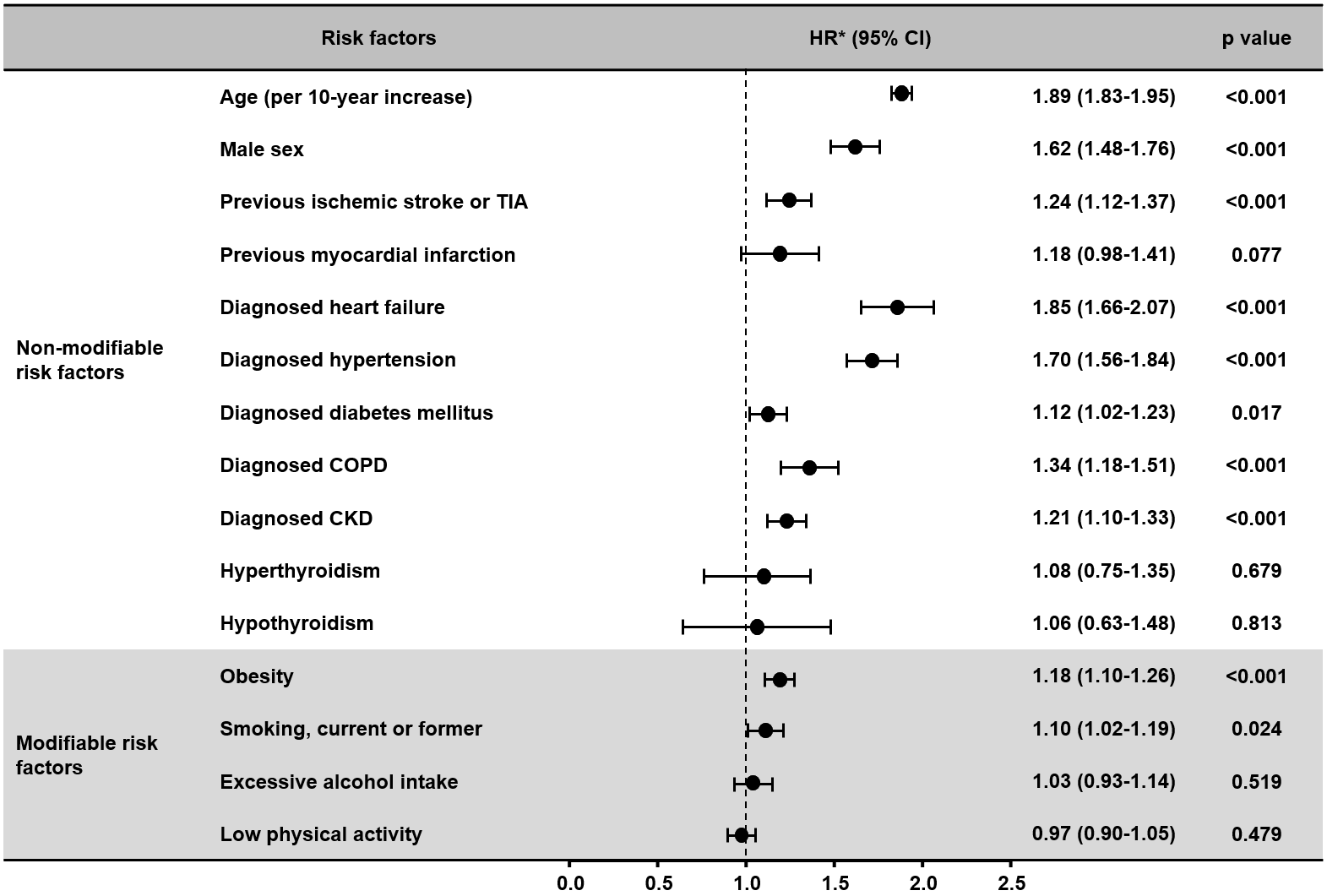


**Supplemental Figure S4.** Subdistribution hazard ratios for new-onset AF according to the risk factors in the overall population. *Subdistribution hazard ratio for each risk factor was adjusted by the Fine and Gray regression model using these variables: age, sex, previous ischemic stroke or TIA, previous myocardial infarction, heart failure, hypertension, diabetes mellitus, COPD, CKD, obesity, smoking, excessive alcohol intake, and low physical activity. CI, confidence interval; CKD, chronic kidney disease; COPD, chronic obstructive pulmonary disease; sHR, subdistribution hazard ratio; TIA, transient ischemic attack.


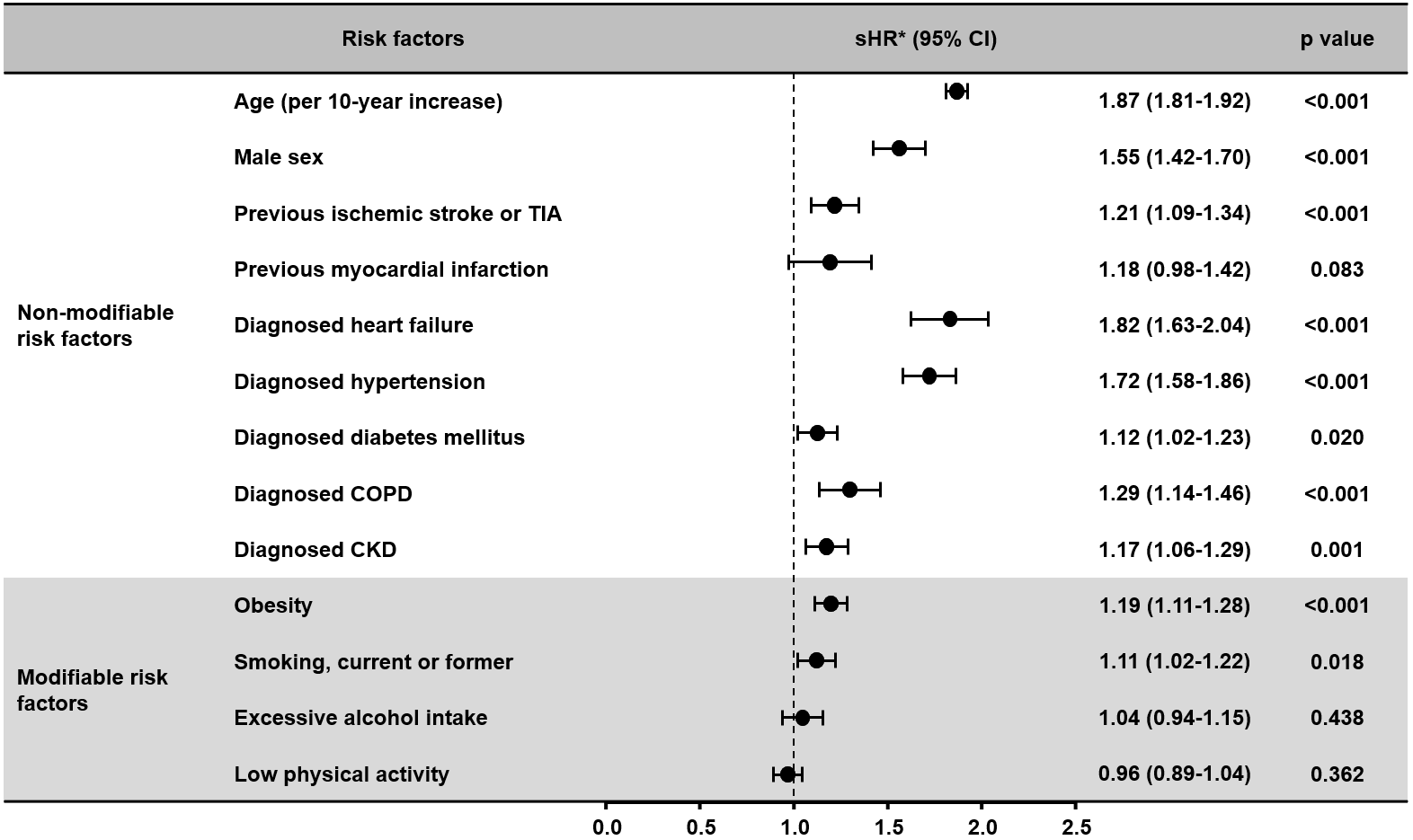


**Supplemental Figure S5.** Subdistribution hazard ratios for new-onset AF according to the risk factors in the “aged <60 years” and “aged ≥60 years” groups. *Subdistribution hazard ratio for each risk factor was adjusted by the Fine and Gray regression model using these variables: age, sex, previous ischemic stroke or TIA, previous myocardial infarction, heart failure, hypertension, diabetes mellitus, COPD, CKD, obesity, smoking, excessive alcohol intake, and low physical activity. # Indicates interaction p-values (<0.05) testing interactions for each risk factor between “aged <60 years” and “aged ≥60 years” groups with adjusting other residual covariables (age, sex, previous ischemic stroke/TIA, previous myocardial infarction, heart failure, hypertension, diabetes mellitus, COPD, CKD, obesity, smoking, excessive alcohol intake, and low physical activity). Among “aged <60 years” group, the risks of incident AF were significantly higher for age (per 10-year increase), hypertension, and obesity variables (each interaction p-value <0.001) compared to those of “aged ≥60 years” group. CI, confidence interval; CKD, chronic kidney disease; COPD, chronic obstructive pulmonary disease; sHR, subdistribution hazard ratio; TIA, transient ischemic attack.

(next page)

**(Supplemental Figure S5)**


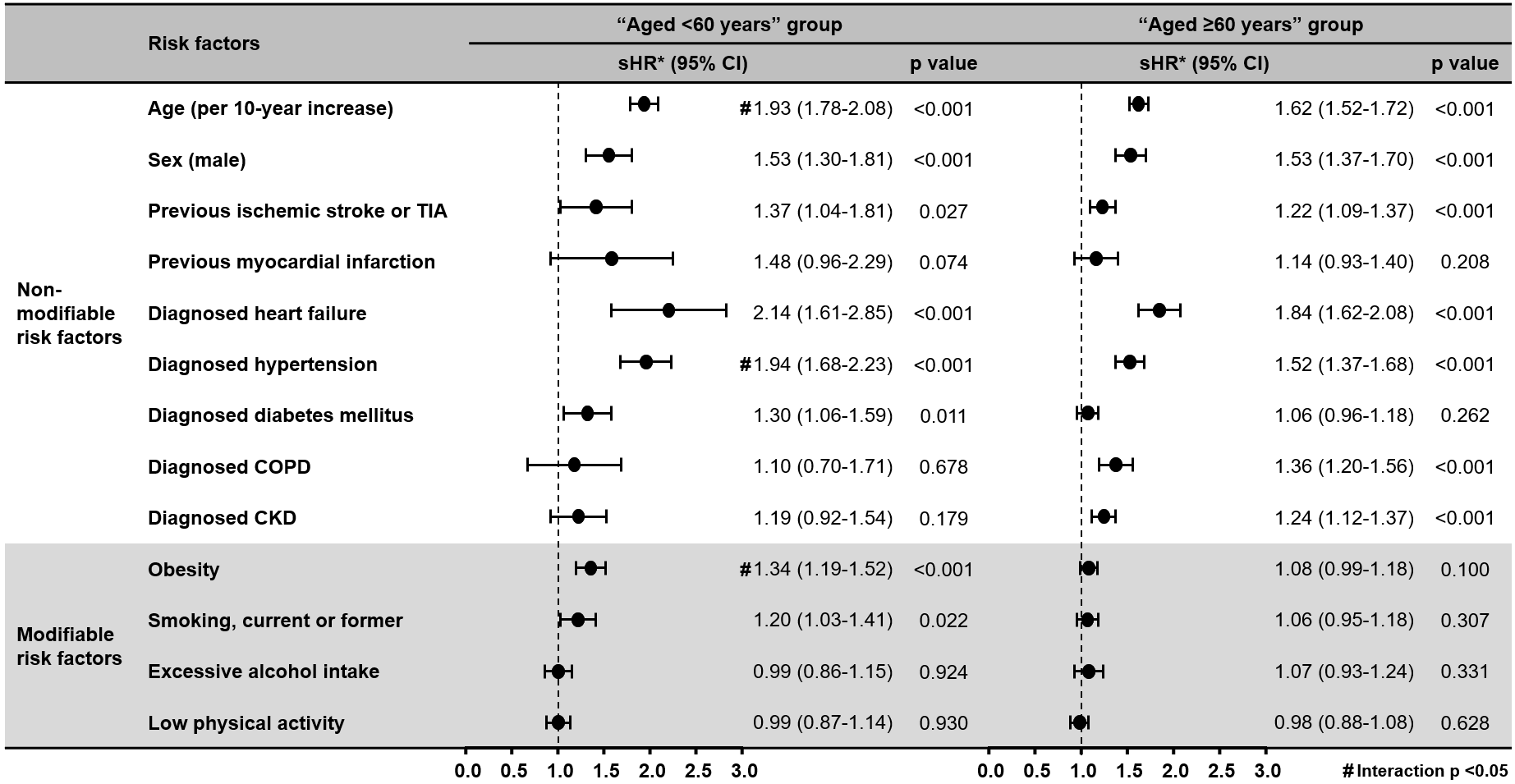


**Supplemental Figure S6.** Alcohol intake (glass/week) and adjusted hazard ratios for new-onset AF in the “aged <60 years” and “aged ≥60 years” groups. Hazard ratios (HR) for alcohol intake was adjusted by the Cox regression model using these variables: age, sex, previous ischemic stroke or transient ischemic attack, previous myocardial infarction, heart failure, hypertension, diabetes mellitus, chronic obstructive pulmonary disease, chronic kidney disease, obesity, smoking, and low physical activity. Color areas are 95% confidence intervals for the spline curves. HR, hazard ratio.


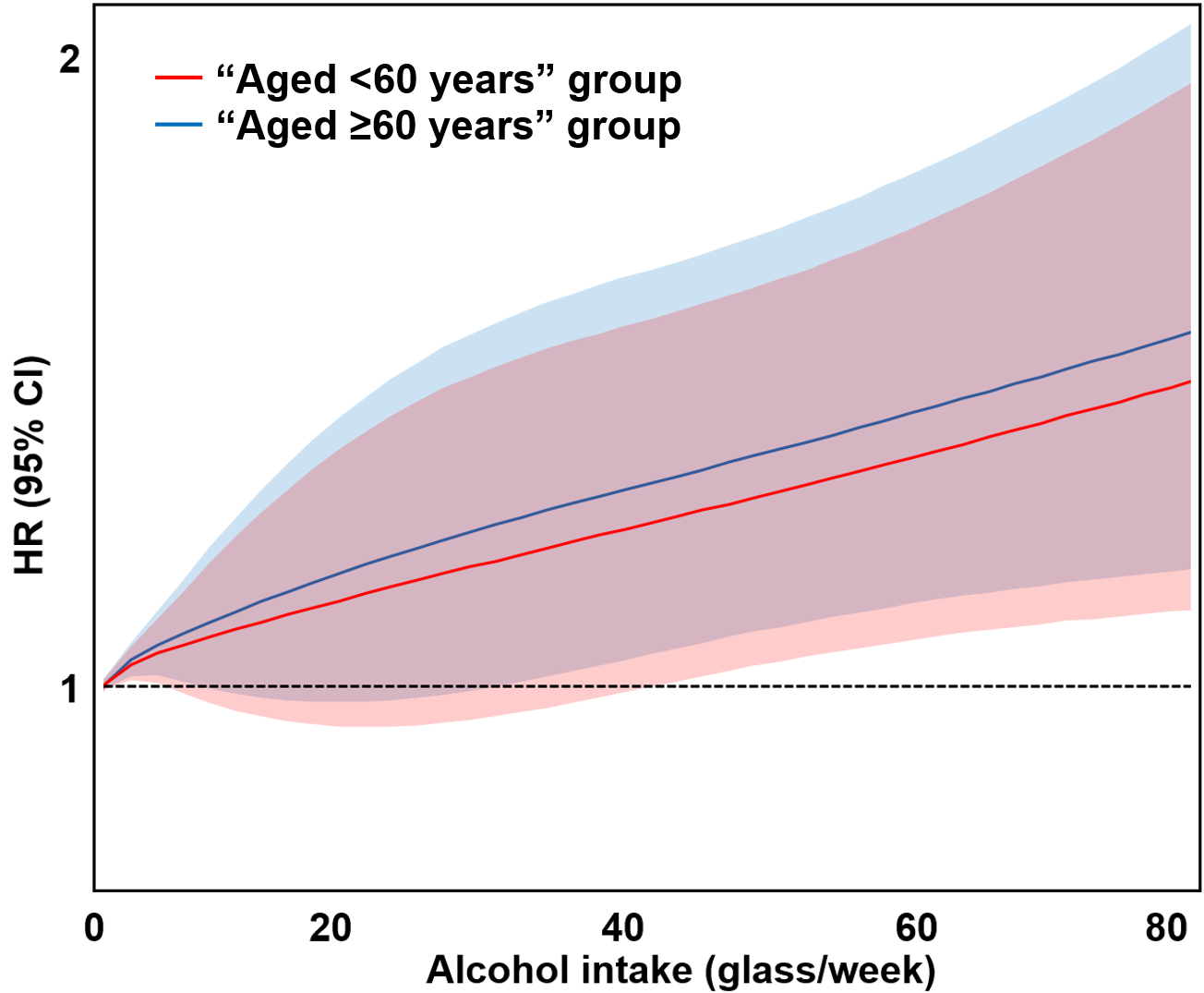


**Supplemental Figure S7.** (A) Body mass index and adjusted subdistribution hazard ratios for new-onset AF in the “aged <60 years” and “aged ≥60 years” groups. (B) Smoking amount (pack-year) and adjusted subdistribution hazard ratios for new-onset AF in the “aged <60 years” and “aged ≥60 years” group. Subdistribution hazard ratio (sHR) for each risk factor was adjusted by the Fine and Gray regression model using these variables: age, sex, previous ischemic stroke or transient ischemic attack, previous myocardial infarction, heart failure, hypertension, diabetes mellitus, chronic obstructive pulmonary disease, chronic kidney disease, obesity, smoking, excessive alcohol intake, and low physical activity (obesity was excluded in the model for [A], smoking status was excluded in the model for [B]). A reference for subdistribution hazard ratio was a BMI of 20 kg/m^2^ in (A) and never smoker in (B). Color areas are 95% confidence intervals for the spline curves. sHR, subdistribution hazard ratio.


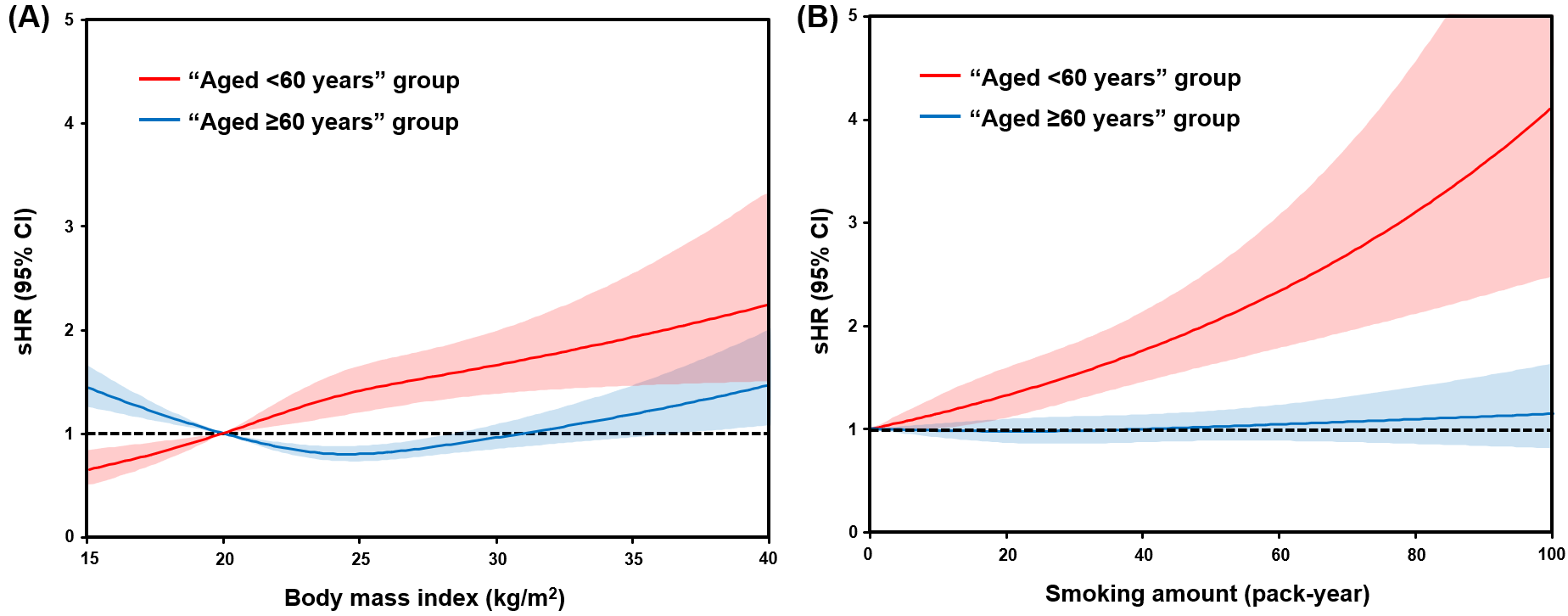


**Supplemental Table S1.** Definitions and ICD-10 codes used for defining the comorbidities and new-onset AF.

| **Comorbidities** | **Definitions** | **ICD-10 codes or conditions** |
| --- | --- | --- |
| Mitral stenosis or prosthetic valve | Defined from any diagnoses of mitral stenosis or heart valve surgery | I05.0, I05.2, I34.2, Z95.2-4, claim for valve replacement or valvuloplasty |
| Heart failure | Defined from diagnosis* | I11.0, I50, I97.1 |
| Hypertension | Defined from diagnosis* | I10, I11, I12, I13, I15 |
| Diabetes mellitus | Defined from diagnosis* plus treatment | E10, E11, E12, E13, E14  Treatment: all kinds of oral antidiabetics and insulin |
| Chronic kidney disease (CKD) | Defined from eGFR (if laboratory value was not available, diagnosis code was used) | eGFR <60 mL/min per 1.73 m^2^  N18, N19 |
| Chronic obstructive pulmonary disease (COPD) | Defined from diagnosis* plus treatment | J42, J43(except J43.0), J44  Treatment: SABA, SAMA, LABA, LAMA, ICS, ICS+LABA, or methylxanthine (>1 month). |
| Dyslipidemia | Defined from diagnosis* | E78 |
| Chronic liver disease | Defined from diagnosis of chronic liver disease, cirrhosis, and hepatitis | B18, K70, K71, K72, K73, K74, K76.1 |
| Hyperthyroidism | Defined from diagnosis* | E05 |
| Hypothyroidism | Defined from diagnosis* | E03 |
| Previous ischemic stroke / transient ischemic attack (TIA) | Defined from diagnosis* | I63, I64, G45 |
| Previous myocardial infarction (MI) | Defined from diagnosis* | I21, I22, I25.2 |
| Cancer | Defined from diagnoses of cancer (non-benign) | C00-C97 |
| New-onset atrial fibrillation (AF) | Defined from diagnosis* without previous insurance claim for AF for 7 years | I48 |

*To ensure accuracy, the diagnosis was established based on one inpatient or two outpatient records of ICD-10 codes in the database.

**Supplemental Table S2.** Sensitivities and specificities of different cutoff levels of age for predicting incident AF.

| **Cutoff level of age** (year) | **Sensitivity** | **Specificity** | **Youden’s index** * |
| --- | --- | --- | --- |
| ≥ 46 | 0.170 | 0.934 | 0.104 |
| ≥ 47 | 0.208 | 0.930 | 0.138 |
| ≥ 48 | 0.246 | 0.931 | 0.177 |
| ≥ 49 | 0.256 | 0.913 | 0.169 |
| ≥ 50 | 0.304 | 0.917 | 0.220 |
| ≥ 51 | 0.318 | 0.913 | 0.230 |
| ≥ 52 | 0.429 | 0.892 | 0.320 |
| ≥ 53 | 0.473 | 0.857 | 0.330 |
| ≥ 54 | 0.486 | 0.849 | 0.335 |
| ≥ 55 | 0.549 | 0.832 | 0.381 |
| ≥ 56 | 0.590 | 0.809 | 0.399 |
| ≥ 57 | 0.646 | 0.792 | 0.439 |
| ≥ 58 | 0.663 | 0.781 | 0.444 |
| ≥ 59 | 0.687 | 0.766 | 0.453 |
| **≥ 60** | **0.717** | **0.740** | **0.457** |
| ≥ 61 | 0.730 | 0.716 | 0.446 |
| ≥ 62 | 0.775 | 0.660 | 0.435 |
| ≥ 63 | 0.791 | 0.578 | 0.369 |
| ≥ 64 | 0.809 | 0.499 | 0.308 |
| ≥ 65 | 0.857 | 0.394 | 0.251 |
| ≥ 66 | 0.871 | 0.318 | 0.189 |
| ≥ 67 | 0.882 | 0.275 | 0.157 |

Bold: the highest Youden’s index

MACE, major adverse cardiovascular event.

* Youden’s index = [ Sensitivity + Specificity - 1 ]

**Supplemental Table S3.** Cox-proportional hazard regression analysis for the incident AF according to the risk factors for the overall population and each age group (eGFR [ml/min], BMI [kg/m^2^], smoking [pack∙year], alcohol intake [g/week], and physical activity habits [hour/week] were analyzed as continuous variables).

| **Variables** | **Overall population** | | **“Aged <60 years” group** | | **“Aged ≥60 years” group** | | **Interaction p-value** # |
| --- | --- | --- | --- | --- | --- | --- | --- |
|  | **Adjusted HR** * **(95% CI)** | **p-value** | **Adjusted HR** * **(95% CI)** | **p-value** | **Adjusted HR** * **(95% CI)** | **p-value** |  |
| **Non-modifiable risk factors** |  |  |  |  |  |  |  |
| Age (per 10-year increase) | **1.87 (1.81-1.93)** | **<0.001** | **1.88 (1.77-2.01)** | **<0.001** | **1.67 (1.59-1.74)** | **<0.001** | **0.003** |
| Male | **1.61 (1.49-1.75)** | **<0.001** | **1.60 (1.38-1.85)** | **<0.001** | **1.59 (1.43-1.75)** | **<0.001** | 0.513 |
| Previous ischemic stroke or TIA | **1.23 (1.10-1.36)** | **<0.001** | **1.37 (1.04-1.81)** | **0.026** | **1.23 (1.10-1.38)** | **<0.001** | 0.334 |
| Previous myocardial infarction | 1.18 (0.98-1.43) | 0.074 | 1.49 (0.97-2.29) | 0.072 | 1.15 (0.94-1.41) | 0.192 | 0.742 |
| Heart failure | **1.84 (1.64-2.06)** | **<0.001** | **2.13 (1.60-2.84)** | **<0.001** | **1.85 (1.64-2.09)** | **<0.001** | 0.682 |
| Hypertension | **1.69 (1.55-1.83)** | **<0.001** | **1.92 (1.67-2.22)** | **<0.001** | **1.51 (1.36-1.66)** | **<0.001** | **<0.001** |
| Diabetes mellitus | **1.12 (1.02-1.24)** | **0.017** | **1.29 (1.06-1.57)** | **0.013** | 1.07 (0.97-1.20) | 0.191 | 0.296 |
| COPD | **1.32 (1.16-1.50)** | **<0.001** | 1.09 (0.70-1.69) | 0.705 | **1.39 (1.22-1.58)** | **<0.001** | 0.633 |
| eGFR (ml/min) | **0.98 (0.97-0.99)** | **<0.001** | 0.99 (0.98-1.01) | 0.099 | **0.98 (0.97-0.99)** | **<0.001** | 0.853 |
| **Modifiable risk factors** |  |  |  |  |  |  |  |
| BMI (kg/m^2^) | **1.03 (1.02-1.04)** | **<0.001** | **1.05 (1.03-1.07)** | **<0.001** | 1.01 (0.99-1.02) | 0.071 | **<0.001** |
| Smoking (pack∙year) | **1.02 (1.01-1.03)** | **0.020** | **1.02 (1.01-1.03)** | **0.030** | 1.01 (0.99-1.02) | 0.355 | 0.061 |
| Alcohol intake (g/week) | 1.01 (0.99-1.03) | 0.300 | 1.01 (0.98-1.03) | 0.700 | 1.01 (0.99-1.04) | 0.297 | 0.747 |
| Physical activity (hour/week) | 1.02 (0.98-1.06) | 0.124 | 1.01 (0.99-1.03) | 0.574 | 1.02 (0.98-1.05) | 0.069 | 0.429 |

AF, atrial fibrillation; BMI, body mass index; CI, confidence interval; CKD, chronic kidney disease; COPD, chronic obstructive pulmonary disease; eGFR, estimated glomerular filtration rate (estimated by serum creatinine using CKD-EPI formula[1]); HR, hazard ratio; TIA, transient ischemic attack.

*Adjusted HR for each risk factor was adjusted by Cox regression model using these variables: age (per 10-year increase), sex, previous ischemic stroke or TIA, previous myocardial infarction, heart failure, hypertension, diabetes mellitus, eGFR, COPD, BMI, smoking, alcohol intake, and physical activity.

# Indicates interaction p-values testing interactions for each risk factor between “aged <60 years” and “aged ≥60 years” groups with adjusting other residual covariables (age, sex, previous ischemic stroke or TIA, previous myocardial infarction, heart failure, hypertension, diabetes mellitus, COPD, eGFR, BMI, smoking, alcohol intake, and physical activity). Among subjects with “aged <60 years”, the risks of incident AF were significantly higher for age (per 10-year increase), hypertension, and BMI variables (each interaction p-value <0.01) compared to those of the “aged ≥60 years” group.

**Supplemental Table S4.** Hazard ratios for new-onset AF according to the risk factors in the overall population (age as a categorical variable: “age ≥60 years or not”).

| **Variables** | **Adjusted HR** * **(95% CI)** | **p-value** |
| --- | --- | --- |
| **Age ≥60 years** | **3.85 (3.54-4.19)** | **<0.001** |
| **Male sex** | **1.51 (1.40-1.64)** | **<0.001** |
| **Previous ischemic stroke/TIA** | **1.34 (1.21-1.48)** | **<0.001** |
| **Previous myocardial infarction** | **1.20 (1.01-1.45)** | **0.048** |
| **Heart failure** | **2.01 (1.79-2.24)** | **<0.001** |
| **Hypertension** | **2.13 (1.96-2.32)** | **<0.001** |
| **Diabetes mellitus** | **1.12 (1.02-1.24)** | **0.017** |
| **COPD** | **1.54 (1.36-1.74)** | **<0.001** |
| **CKD** | **1.45 (1.32-1.59)** | **<0.001** |
| **Obesity** | **1.10 (1.02-1.18)** | **0.009** |
| **Smoking, current or former** | **1.02 (1.01-1.03)** | **<0.001** |
| **Excessive alcohol intake** | 0.97 (0.87-1.07) | 0.526 |
| **Low physical activity** | 1.01 (0.93-1.09) | 0.835 |

CI, confidence interval; CKD, chronic kidney disease; COPD, chronic obstructive pulmonary disease; HR, hazard ratio; TIA, transient ischemic attack.

* Hazard ratio (HR) for each risk factor was adjusted by the Cox regression model using these variables: age ≥60 years (age as a categorical variable), sex, previous ischemic stroke or TIA, previous myocardial infarction, heart failure, hypertension, diabetes mellitus, COPD, CKD, obesity, smoking, excessive alcohol intake, and low physical activity.
